# Examining the Relation Between Practicing Meditation and Having Peak Experiences and Lucid Dreams. A Cross-Sectional Study

**DOI:** 10.3389/fpsyg.2022.858745

**Published:** 2022-04-26

**Authors:** Javier Garcia-Campayo, Rinchen Hijar-Aguinaga, Alberto Barceló-Soler, Selene Fernández-Martínez, Roberto Aristegui, Adrián Pérez-Aranda

**Affiliations:** ^1^Institute of Health Research of Aragon (IIS), Zaragoza, Spain; ^2^Department of Medicine, Psychiatry and Dermatology, Faculty of Medicine, University of Zaragoza, Zaragoza, Spain; ^3^Navarra Medical Research Institute (IdiSNA), Pamplona, Spain; ^4^Department of Psychiatry and Mental Health Oriente, Faculty of Medicine, University of Chile, Santiago, Chile; ^5^Department of Basic, Developmental and Educational Psychology, Autonomous University of Barcelona, Cerdanyola del Vallès, Spain

**Keywords:** mindfulness, peak experience, lucid dreams, absorption, non-dual experience, self-transcendence

## Abstract

The aim of this study was to compare meditators and non-meditators in terms of their tendency to have peak experiences and their dream lucidity, while examining the associations between these outcomes and some related variables such as non-dual awareness, mindfulness facets and absorption. In this cross-sectional study, 237 participants from general Spanish population completed an online survey that included *ad hoc* questions related to the study aim, along with the Five Facet Mindfulness Questionnaire (FFMQ), the Non-dual Embodiment Thematic Inventory (NETI), the Tellegen Absorption Scale (TAS) and the Lucidity and Consciousness in Dreams Scale (LUCID). Of the total, 110 participants were identified as meditators and 127 as non-meditators. More than half of the sample (58.2%) reported having experienced at least one peak experience in their life; these showed no differences in the number, intensity, or self-inducing ability of these experiences between both groups but were significantly more common among meditators (71.8% vs. 46.8%; *p* < 0.001), who also presented higher scores in most of the questionnaires, except for some LUCID subscales. Regression models demonstrated that being a meditator was a significant predictor of having had a peak experience, but not of LUCID scores. These results, which need to be interpreted considering the study limitations, support the potential of meditation to facilitate having peak experiences, while its impact on lucid dreams remains unclear.

## Introduction

Meditation has become a normalized activity for many people, regardless of whether it is practiced as a technique for reducing stress or as a contemplative exercise in a spiritual or religious context. In recent decades, several studies have observed that individuals who meditate regularly present significant differences when compared to non-meditators, including greater self-acceptance and self-awareness, feeling less upset over criticism from others, less somatic symptomatology such as pain, better health (particularly, mental health) and different health-related behaviors (e.g., lower use of tranquilizers or drugs to change mood) (Easterlin and Cardeña, 1998; Monk-Turner, 2003; Manocha et al., 2012); even brain function during attention tasks and heart rate variability dynamics have been observed to be different in meditators (Kozasa et al., 2018; Goshvarpour and Goshvarpour, 2019; Luders and Kurth, 2019). These differences have been attributed by some authors to certain differential mechanisms related to attention processing, as well as neuroplasticity, which are activated through the regular practice of contemplative exercises in which self-awareness plays a key role (Kozasa et al., 2018; Guidotti et al., 2021).

Moreover, different types of meditation practices have proven effective for improving the symptomatology of many syndromes and conditions. Among the different meditation programs that have been tested, those based on the systematic practice of mindfulness (i.e., present-focused, non-judgmental awareness) have shown to be particularly effective for treating different conditions, including depressive, anxiety, stress-related, and hyperactivity disorders, as well as headaches and cardiovascular and neurodegenerative diseases, among others (Chimiklis et al., 2018; Chételat et al., 2018; Goldberg et al., 2018; Gu et al., 2018; Scott-Sheldon et al., 2020).

However, little study has been given to the potential of practicing meditation techniques to promote wellbeing through other ways that do not imply reducing pathological symptomatology. In this regard, transcendent experiences, defined as those of a state of consciousness characterized by altered or expanded awareness (Levin and Steele, 2005), can represent a source of wellbeing, and the upsurge in contemplative sciences in recent years has increased the interest shown in such phenomena (Garcia-Campayo et al., 2021). Peak experiences and lucid dreams are two examples of transcendent experiences that could be enhanced, both in terms of frequency and intensity, through the practice of meditation.

The term “peak experience” was used by psychologist Abraham Maslow to describe states of unitive consciousness, or “moments of highest happiness and fulfillment” in people’s lives (Maslow, 1964). Many other terms have been used to describe the same phenomenon, including non-dual awareness, enlightenment, mystical experience, and transcendental experience (Martin et al., 2021). At such times, individuals feel detached from the mundane particulars of their individual lives and sense that they are at one with a fully integrated universe. The emotions frequently reported during peak experiences include wonder, awe, reverence, humility, surrender, and even worship before the greatness of the experience (Maslow, 1964).

Achieving a peak experience is the ultimate goal of many traditional meditation techniques; however, Western science has focused much more on the long-term trait effects of meditation, achieved after years of training, that are thought to be therapeutic (Rubia, 2009). Although the impact of meditation trainings on the frequency or intensity of peak experiences has not been widely studied, some studies have analyzed the effects of meditative techniques on the development of non-dual awareness. A study conducted by Mills et al. (2018) found that an intensive 6-day mind-body program addressed at physical, emotional, and spiritual domains was effective for increasing the levels of non-dual embodiment and that these changes were maintained after 1 month. In the same vein, Martin et al. (2021), who deepened into the so-called “plateau experiences”—also related to altered self-transcendence but in a more calm, persistent (i.e., effects lasting for at least 1 year) way compared to peak experiences (Krippner, 1972)–, reported that most of the participants in their studies transitioned to deeper states of self-transcendence after taking part in online meditation programs that included positive psychology exercises (e.g., forgiveness, gratitude exercises). The self-transcendent state of the participant and the possible transition to deeper states were based on self-reported measures and semi-structured interviews conducted by an expert in the field. This study included many relevant variables, such as non-dual awareness, which is defined as an open, awake cognizance that goes beyond the primary identification with the mind–body and transcends the subject–object dichotomy identification to where ordinary distinctions of subject and object do not exist (Mills et al., 2018). However, these studies did not quantify the frequency nor the intensity of peak experiences, nor its association with other key variables such as mindfulness facets or absorption.

Mindfulness has been described as a multifactorial construct that includes the facets of observing (i.e., noticing or attending to internal experiences such as sensations, thoughts, or emotions), describing (i.e., labeling thoughts and emotions with words), acting with awareness (i.e., focusing on one’s activities in the moment as opposed to behaving mechanically), non-judging of the inner experience (i.e., taking a non-evaluative stance toward thoughts and feelings), and non-reacting to the inner experience (i.e., allowing thoughts and feelings to come and go, without getting caught up in or carried away by them) (Baer et al., 2008). While dispositional mindfulness has been associated with non-dual awareness (Hanley et al., 2018), and therefore the possibility of having peak experiences, the role of the mindfulness facets has not yet been explored. Absorption, on the other hand, is defined as a disposition for having episodes of total attention that result in a heightened sense of the reality of the attentional object, imperviousness to distracting events, and an altered sense of reality in general (Tellegen and Atkinson, 1974). The connection between absorption, mindfulness and peak experiences is conceptually clear and previous studies have already reported higher levels of trait absorption in meditators (Holzel and Ott, 2006; Bowden et al., 2012; Grant et al., 2013), and Berkovich-Ohana and Glicksohn (2017) observed that trait absorption is associated with mindfulness expertise and with transcendent experience.

In another vein, lucid dreaming, which is defined as the experience of knowing one is dreaming while one is dreaming (LaBerge, 1985), has also been described as a transcendent experience linked to wellbeing, increased self-confidence, psychological resilience, and positive emotions (Soffer-Dudek, 2020). Lucid dreaming is considered a hybrid state of consciousness in which part of the brain operates in the primary mode, while another part has access to secondary consciousness (Stumbrys et al., 2012; Voss et al., 2013). Research indicates that around 55% of adults have experienced at least one lucid dream, while 23% experience them frequently (Saunders et al., 2016). Previous research has already reported significant links between absorption and lucid dreaming—as well as with other consciousness states—suggesting that it could be considered a predisposing factor for such experience (Glicksohn and Barrett, 2003; Hunt, 2007; Terhune, 2009; Rosen et al., 2017; Glicksohn, 2019). In addition to absorption, some authors have identified other variables associated to that lucid dreaming that suggest that this experience can be somehow trained; in this regard, Stumbrys and Erlacher (2017) concluded that cultivating mindfulness could increase dream control in those who experience lucid dreams. Interestingly, dispositional mindfulness in wakefulness has been associated with lucid dream frequency (Stumbrys et al., 2015), suggesting that the attentional control mechanisms that underline mindfulness trait could be also active in the dream state, increasing the ability to control the dream. However, longitudinal studies should be conducted to overcome the confirm such hypotheses.

The main objective of the present study was to explore the associations between practicing meditation and the transcendent experiences of peak experiences and lucid dreams in the general population. The role of non-dual awareness, mindfulness facets, and absorption was also assessed as possible predictors of the individual’s tendency to present peak experiences and dream lucidity. The authors’ hypotheses were that the frequency and intensity of peak experiences and the occurrence of dream lucidity would be significantly higher among meditators, and that non-dual awareness, absorption, and mindfulness facets would be significant predictors of the primary outcomes.

## Materials and Methods

### Participants and Procedure

Participants were individuals who had taken the Master of Mindfulness program at the University of Zaragoza (Spain) and students from other courses on mindfulness and related disciplines offered by our group. The recruitment was conducted from February through April, 2021; emails with information regarding the study were sent to every potential candidate. Those who agreed to participate by signing the informed consent were provided with a link to the survey, which, in addition to questions pertaining to sociodemographic information and validated questionnaires, contained a number of *ad hoc* questions on peak experiences and meditation practice (see section “Instruments”). SurveyMonkey was the platform used for collecting the data.

The study protocol was approved by the ethical review board of the regional health authority of Aragon, Spain (PI21/243). All procedures performed in this study were in accordance with the 1964 Helsinki Declaration and its later amendments.

### Instruments

The online survey included *ad hoc* questions regarding sociodemographic information (gender, age, nationality, civil status, level of studies, employment status), meditation (experience, frequency, minutes of practice, years of practice, context of meditation), and peak experiences (experience, frequency, intensity, consequences, ability to self-induce the peak experience, and the feelings derived from it). The following questionnaires were also included to assess the main constructs of the present study:

The *Five Facets of Mindfulness Questionnaire*, 24-item version (FFMQ-24; Bohlmeijer et al., 2011) is a short version of the original FFMQ (Baer et al., 2008) in which the five facets of mindfulness are assessed: “observing” (4 items), “describing” (5 items), “acting with awareness” (5 items), “non-judging” (5 items), and “non-reacting to the inner experience” (5 items). The score of the subscales ranges between 5 and 25, except for “observing,” which ranges between 4 and 20. The total score of the FFMQ-24 is calculated by adding up the scores of the subscales and ranges between 24 and 120. Higher scores indicate higher levels of mindfulness. The FFMQ-24 presents strong psychometric properties, similar to the ones of the original FFMQ (Bohlmeijer et al., 2011). The scale was validated in Spanish by Asensio-Martínez et al. (2019) with internal consistency ranging between α = 0.65 (“observing”) and 0.80 (“acting with awareness”). The internal consistency of the total scale in the present study was high (α = 0.88, subscales ranging between 0.72, for “observing,” and 0.86 for “non-judging”).

The *Non-dual Embodiment Thematic Inventory* (NETI; Butlein, 2006) is a 20-item questionnaire designed to evaluate qualities of the non-dual experience and spiritual awakening. NETI assesses a number of variables, including frequency of non-dual experiences, which refer to states of self-transcendence states that have also been referred to as peak experiences (Martin et al., 2021). The total score is calculated with a range between 20 and 100, with higher scores indicating higher non-dual awareness. The scale has shown good psychometric properties (α = 0.91) (Butlein, 2006; Martin et al., 2021) and was translated to Spanish for the purpose of this study. The internal consistency of the NETI in the present study was high (α = 0.81).

The *Tellegen Absorption Scale* (TAS; Tellegen and Atkinson, 1974) is a 34-item questionnaire that assesses absorption, which has been previously described (section “Introduction”) as a construct closely related to hypnotizability, openness to experience, and imagination (Glisky and Kihlstrom, 1993). Moreover, absorption is associated with periods of relaxed focused attention (Owens et al., 1999). Each item requires a dichotomous answer (Yes/No), and every positive answer is added up to give a total score that can range between 0 and 34, where higher scores indicate higher levels of absorption. TAS has shown good psychometric properties (Kihlstrom et al., 1989) and was translated to Spanish by Perona-Garcelán et al. (2013) with high internal consistency (α = 0.93), similar to the one in the present study (α = 0.88).

The *Lucidity and Consciousness in Dreams Scale* (LUCID; Voss et al., 2013) is a 28-item questionnaire that assesses key aspects of dream lucidity in detail regarding one recent dream that the respondent has to choose. LUCID presents 8 subscales: insight, control, thought, realism, memory, dissociation, negative emotion, and positive emotion. Each item is answered using a 6-point Likert scale (0 = never, 5 = always); a score for every subscale as well as a global score can be obtained by calculating the mean of the items. Scores range between 0 and 5, with higher scores indicating higher levels of lucidity. The Spanish version of LUCID has presented good psychometric properties (α ranging between 0.65 for “memory” and 0.83 for positive emotion) (García-Campayo et al., 2021). The internal consistency of the total scale in the present study was very high (α = 0.91, subscales ranging between 0.72, for “realism” and “dissociation,” and 0.90 for “positive emotion”).

### Analysis

The sociodemographic characteristics and study variables were described using frequencies (and percentages) or means (and standard deviation, SD), depending on the distribution of each variable.

Two subgroups were defined based on the self-reported experience of meditation and frequency of practice: those who reported practicing meditation at least once per month were considered meditators, while those who reported no experience with it or practicing less than once per month were considered non-meditators. Chi-squared or Student’s *t*-tests were conducted to assess between-group differences. Bivariate correlations between the study variables were computed and reported as Supplementary Material.

Linear regression models were computed to analyze the predictive role of sociodemographic (i.e., sex, age, level of education, civil status, and employment status) and study variables (i.e., being a regular meditator and the scores of the questionnaires and its subscales) on FFMQ, NETI, TAS, and LUCID total scores. A logistic regression model was computed to analyze the predictive role of the same variables on the self-reported occurrence of a peak experience.

An alpha level of.050 was set, using a two-tailed test. The Benjamini-Hochberg correction for multiple tests was applied (Glickman et al., 2014). Data analyses were computed using IBM SPSS v26.0 statistical software.

## Results

### Participants’ Sociodemographic Characteristics

A total of 237 individuals participated in the present study. Most of them were females (*n* = 178, 75.1%) and Spanish (*n* = 199, 84%), with a mean age of 44.30 years (*SD* = 13.00). Approximately half of the participants were married or in a stable relationship (*n* = 131, 55.3%), most had finished university studies (*n* = 184, 77.9%), and were in employment (*n* = 174, 73.4%). Table 1 provides the participants’ sociodemographic characteristics in detail.

**TABLE 1:** Baseline characteristics of patients by treatment group.

	Meditators (*n* = 110)	Non-meditators (*n* = 127)	Total (*n* = 237)	*p*
Age, mean *(SD)*	44.78 (11.64)	43.89 (14.11)	44.30 (13.00)	0.873
Gender, *n of females (%)*	90(81.80%)	88(69.3%)	178(75.1%)	**0.026**
**Marital status, *n (%)***				
Single	35(31.8%)	43(33.9%)	78(32.9%)	0.333
Married/relationship	59(53.6%)	72(56.7%)	131(55.3%)	
Separated/divorced	15(13.6%)	9(7.1%)	24(10.1%)	
Widowed	1(0.9%)	3(2.4%)	4(1.7%)	
**Nationality, *n (%)***				
Spanish	89(80.9%)	110(86.6%)	199(84%)	0.242
Others (Latin-American)	17(15.5%)	16(12.6%)	33(13.9%)	
Others (European)	4(3.6%)	1(0.8%)	5(2.1%)	
**Education, *n (%)***				
No studies	0(0%)	1(0.8%)	1(0.4%)	**0.040**
Primary studies	2(1.8%)	8(6.3%)	10(4.2%)	
Secondary studies	13(11.9%)	28(22%)	41(17.4%)	
University	94(86.2%)	90(70.9%)	184(77.9%)	
**Employment, *n (%)***				
Employed	83(75.5%)	91(71.7%)	174(73.4%)	0.136
Unemployed	11(10%)	7(5.5%)	18(7.5%)	
Home duties	3(2.7%)	1(0.8%)	4(1.7%)	
Student	3(2.7%)	10(7.9%)	13(5.5%)	
Sick leave	5(4.5%)	4(3.2%)	9(3.8%)	
Retired	5(4.5%)	14(11%)	19(8%)	

*In bold, statistically significant results.*

### Meditation Experience

According to the self-reported answers given by the study participants, 113 (47.7%) of them had experience in practicing meditation, while the remaining 124 did not. With regard to frequency of practice, 28 people (11.8%) reported practicing meditation daily, 36 (15.2%) did so multiple times per week, 20 (8.4%) practiced once per week, 26 (10.7%) practiced meditation at least once per month, and 3 (1.3%) never practiced. The mean practice duration in minutes was 22.46 (*SD* = 13.84).

Among those who had experience with meditation, 63 (55.8%) had learned by taking a course, 13 (11.5%) had learned in a religious context, 10 (8.8%) had learned in the context of therapy, and 26 (23%) were self-taught. Of these, 21 participants (18.6%) reported having been practicing for less than a year, while most had between 1 and 3 years of practice (*n* = 36, 31.9%), and 20 (17.7%) had more than 10 years’ experience. Only 15 participants (13.3%) used meditation in a religious context.

This information was used to create two subgroups based not only on experience with meditation but also on the reporting of at least sporadic frequency of practice: meditators (i.e., those who reported practicing meditation at least once per month; *n* = 110) and non-meditators (i.e., those who reported no experience with meditation or practiced less than once per month; *n* = 127). Table 1 shows the characteristics of each subgroup; some significant differences were appreciated in the variables “Gender” and “Education level” (*p* < 0.050), since greater proportions of females and higher education levels were observed among meditators.

### Peak Experiences

More than half of the sample (*n* = 138, 58.2%) reported having experienced at least one peak experience in their life, with a mean of 13.68 (*SD* = 29.35), although two subjects that were not included in this analysis reported having experienced countless peak experiences. The mean intensity (0–100) of these experiences was 68.81 (*SD* = 28.02), although it should be noted that 52 individuals (37.7%) described an intensity of 80 or higher.

The mean for the number of participants who reported that peak experiences involved a change in their lives (0–100) was 58.72 (*SD* = 37.52), and a notable proportion of them (*n* = 48, 34.8%) also reported an impact of 80 or more. These changes could be related to meaning of life (*n* = 79, 57.2%), social relationships (*n* = 69, 50%), emotion regulation (*n* = 68, 49.3%), and spirituality (*n* = 57, 41.3%). Among those who had been through a peak experience, some mentioned feeling complete attention to the present moment (*n* = 72, 52.2%), intense positive affect (*n* = 95, 68.8%), admiration and humility (*n* = 68, 49.3%), self-transcendence (*n* = 56, 40.6%), disorientation (*n* = 54, 39.1%), and a complete loss of fear (*n* = 74, 53.6%). The most predominant of these feelings was positive affect, as reported by 41 of these participants (29.7%).

Only 32 individuals (23.2% of the subsample of participants with peak experiences) reported being able to self-induce one of these experiences. Some of them (*n* = 11) described meditation or other contemplative techniques as potential inducers of peak experiences, while a few referred to nature, music, and drugs. The proportion of individuals who were able to self-induce peak experiences was not significantly different in the two subgroups (χ^2^ = 2.15, *p* = 0.142).

Peak experiences were more common among meditators: 79 of these individuals (71.8%) reported having undergone at least one peak experience, compared with non-meditators (*n* = 46, 46.8%; χ^2^ = 15.11, *p* < 0.001). Nonetheless, there were no differences regarding the number (*t* = 0.81, *p* = 0.421) nor the intensity (*t* = 0.30, *p* = 0.763) of peak experiences between meditators and non-meditators.

Besides practicing meditation, a number of other significant differences were found when comparing those who had at least one self-reported peak experience with those who did not: the only sociodemographic variable that presented a significant difference was the employment status (χ^2^ = 22.69, *p* = 0.004), in which higher proportions of individuals on sick leave and unemployed, and lower proportions of students and retired participants were found among those with at least one self-reported peak experience. A number of scales and subscales presented significantly higher scores in this subgroup: NETI (*t* = −3.98, *p* < 0.001), TAS (*t* = −4.99, *p* < 0.001), FFMQ total score (*t* = −2.54, *p* = 0.012) and its subscales “observing” (*t* = −4.05, *p* < 0.001) and “describing” (*t* = −2.37, *p* = 0.019), and LUCID total score (*t* = −2.50, *p* = 0.013) and its subscales “thought” (*t* = −2.59, *p* = 0.010), “realism” (*t* = −2.55, *p* = 0.011), and “positive emotions” (*t* = −2.47, *p* = 0.014).

### Between-Group Differences in the Study Variables (Meditators vs. Non-meditators)

Table 2 presents the between-group differences in the study variables. Significantly higher scores were observed in FFMQ total score and its subscales for meditators, except for “acting with awareness” (*t* = 1.69, *p* = 0.093). NETI and TAS also presented significant differences, always reflecting higher scores for the meditators. With regard to LUCID, differences were observed in the “thought” and “dissociation” subscales, but these resulted only tendencies close to significance after applying the Benjamini-Hochberg correction.

**TABLE 2:** Between-group differences (i.e., meditators vs. non-meditators) in the study variables.

	Meditators (*n* = 110)	Non-meditators (*n* = 127)	*p*
*FFMQ total, *mean (SD)*	84.75 (12.21)	77.39 (11.34)	**<0.001**
*Observing	15.53 (2.64)	13.83 (2.97)	**<0.001**
*Describing	18.82 (3.91)	17.10 (3.79)	**0.001**
*Acting with awareness	16.42 (3.56)	15.60 (3.87)	0.093
*Non-judging	17.29 (4.11)	15.69 (4.15)	**0.003**
*Non-reacting	16.70 (3.37)	15.17 (2.93)	**<0.001**
*NETI, *mean (SD)*	64.08 (8.42)	59.93 (7.59)	**<0.001**
*TAS, *mean (SD)*	22.09 (6.72)	18.42 (6.82)	**<0.001**
*LUCID total, *mean (SD)*	1.96 (0.90)	1.86 (0.84)	0.352
Insight	1.64 (1.26)	1.72 (1.15)	0.582
*Control	1.55 (1.36)	1.38 (1.19)	0.315
*Thought	2.36 (1.47)	1.95 (1.43)	0.029
*Realism	2.95 (1.29)	2.84 (1.31)	0.532
*Memory	1.73 (1.33)	1.64 (1.20)	0.576
*Dissociation	1.53 (1.33)	1.16 (1.24)	0.027
*Negative emotion	1.78 (1.49)	1.99 (1.49)	0.280
*Positive emotion	2.15 (1.66)	2.16 (1.55)	0.956

*In bold, statistically significant results, even after applying the Benjamini-Hochberg correction. The differences in “Realism” and “Dissociation” (LUCID) were not statistically significant after applying the correction (i.e., p > 0.050).*

The bivariate correlations between the study variables are reported as Supplementary Material. As expected, significant positive correlations were found between the FFMQ total score (and most of its subscales), the NETI and the TAS. These two latter scales also correlated positively and significantly with the LUCID total score, while only some significant associations were found with the LUCID subscales.

### Predictive Role of the Study Variables in Self-Reported Peak Experiences, Levels of Mindfulness, Non-dual Transcendence, Absorption, and Lucidity in Dreams

A binary logistic regression was computed considering having undergone a peak experience as the outcome. A significant model (χ^2^ = 36.14, *R*^2^ = 0.19, *p* < 0.001) showed that peak experiences were predicted by higher scores in the TAS (*B* = 0.08, *p* < 0.001) and in the LUCID-Realism subscale (*B* = 0.24, *p* > 0.036), and by being a meditator (*B* = 0.85, *p* = 0.004).

In the case of non-dual transcendence (i.e., NETI score), the linear regression model (*F* = 43.86, *R*^2^ = 0.49, *p* < 0.001) showed as significant predictors the following variables, all of them with a positive influence: FFMQ-Non-reacting (*B* = 1.11, *t* = 8.00, *p* < 0.001), FFMQ-Non-judging (*B* = 0.49, *t* = 4.81, *p* < 0.001), TAS (*B* = 0.28, *t* = 4.81, *p* < 0.001), the LUCID-Positive emotion subscale (*B* = 0.52, *t* = 2.13, *p* = 0.034) and the employment status (*B* = 0.51, *t* = 2.05, *p* = 0.041).

A significant linear regression model (*F* = 30.28, *R*^2^ = 0.44, *p* < 0.001) indicated that the levels of mindfulness (i.e., FFMQ total score) were positively predicted by NETI (*B* = 0.76, *t* = 9.29, *p* < 0.001), TAS score (*B* = 0.23, *t* = 2.36, *p* = 0.019), the LUCID subscale “realism” (*B* = 1.50, *t* = 2.99, *p* = 0.003) and being a meditator (*B* = 2.87, *t* = 2.33, *p* = 0.026), and negatively predicted by the LUCID subscales of “negative emotions” (*B* = −1.53, *t* = −3.68, *p* = 0.001) and “positive emotions” score (*B* = −1.46, *t* = −3.51, *p* = 0.001).

Absorption levels (i.e., TAS scores) were significantly predicted (*F* = 47.94, *R*^2^ = 0.44, *p* < 0.001) by the following variables: FFMQ-Observing (*B* = 1.22, *t* = 10.04, *p* < 0.001), NETI (*B* = 0.21, *t* = 4.38, *p* < 0.001), and LUCID (*B* = 1.54, *t* = 3.77, *p* < 0.001) had a positive influence in the predictive model, while FFMQ-Non-judging (*B* = −0.31, *t* = −3.38, *p* = 0.001) had a negative one.

A significant linear regression model (*F* = 13.63, *R*^2^ = 0.15, *p* < 0.001) showed that the increase in LUCID scores was positively predicted by TAS score (*B* = 0.04, *t* = 5.54, *p* < 0.001) and negatively predicted by age (*B* = −0.01, *t* = −2.97, *p* = 0.003) and education level (*B* = −0.20, *t* = −2.35, *p* = 0.019).

## Discussion

This study explored the associations between practicing meditation and having the transcendent experiences of peak experiences and lucid dreaming, while studying the possible predictive role of the key variables of non-dual awareness, mindfulness facets, and absorption on the study outcomes.

Participants were divided according to their self-reported experience and frequency of practicing meditation into two groups (meditators and non-meditators). Two statistically significant differences were found between the two groups: there was a higher proportion of females and individuals with university education among meditators. Despite being minor, these differences can be important since it has been described that transcendent experiences can be more frequent among females and people with higher education (Hay and Morisy, 1978). In our study, close to 60% of the total sample reported having undergone at least one peak experience in their life, which represents a notably higher proportion than those reported in previous studies conducted some decades ago in the United Kingdom and the United States (Glock and Stark, 1965; Greeley, 1975; Hay and Morisy, 1978). A more recent study (Hoffman et al., 2012a) asked 153 Americans aged 40–65 to describe a recent peak experience, in this case simply defined as a recent joyful experience; while most referred to an interpersonal experience (e.g., becoming a father), only six people (3.9%) reported a religious or philosophical experience, which would be closer to the concept of peak experience that has been used in the present study. More studies have focused on exploring peak experiences during childhood and youth, finding that this phenomenon is common and transcultural (Hoffman and Muramoto, 2007; Hoffman et al., 2010, 2012b).

Our study adds information regarding the characteristics of the peak experiences that previous studies had not reported: the mean number of peak experiences was 13.68 and the mean intensity (0–100) was 68.81, while the mean impact on the individual’s life (0–100) was 58.72. This impact was mainly observed in terms of meaning of life, and intense positive affect was commonly felt during peak experience. These results are in line with the classic characterization of transcendent experiences, defined by fulfillment (i.e., generation of positive emotions and the intrinsic rewarding nature of the experience), significance (i.e., increased personal understanding derived from the experience) and spirituality (i.e., the feeling of being one with the world) (Privette, 2001).

As hypothesized, the proportion of individuals who reported having at least one peak experience was significantly higher among participants who practiced meditation: 71.8% compared with 46.8% of non-meditators. This significant difference supports the idea that practicing meditation can promote transcendent experiences, which was the original aim of many meditation traditions (Rubia, 2009). The practice of contemplative techniques, such as mindfulness, can help individuals to develop non-dual awareness, which is a key element of transcendent experiences since these imply a state of consciousness characterized by altered or expanded awareness (Levin and Steele, 2005). This was observed in our study when the subgroup of meditators presented significantly higher NETI scores than non-meditators. Moreover, and again as expected, individuals who practiced meditation showed significantly higher levels of mindfulness, including all the mindfulness facets, except “acting with awareness,” which only presented a tendency close to significance. Likewise, absorption levels measured by TAS were significantly higher among those who practiced meditation.

Nonetheless, significant differences between meditators and non-meditators were not found for some of the assessed variables related to peak experiences. That was the case of the number of peak experiences in a lifetime, and their mean intensity. This could suggest that these variables may not be directly associated with the practice of meditation but with some other characteristics. In this regard, our study showed significantly higher scores for non-dual awareness, absorption, and the mindfulness facets of observing and describing, together with a number of LUCID subscales, being achieved by individuals who had at least one peak experience compared to those who had never had one. Some of these variables seem to be particularly related to the experience of transcendent states (Owens et al., 1999; Hanley et al., 2018; Martin et al., 2021). However, while the practice of meditation is associated with increases in these outcomes, some individuals may already present high levels in these areas without the need to practice mindfulness or other contemplative techniques, possibly allowing them to achieve the same number and intensity of peak experiences.

Another variable that did not show significant differences between meditators and non-meditators was the proportion of individuals who reported being able to self-induce a peak experience. In total, 32 individuals said that they could induce one of these experiences via different means (e.g., meditation, contact with nature, music, drugs). Interestingly, Maslow (1964) considered that peak experiences could not be self-induced, which could indicate that these participants may not have shared his understanding of the term “peak experience” in the same way, although he did acknowledge that some forms of art could be triggers for transcendent experiences.

As far as dream lucidity is concerned, two tendencies close to significance were observed in the two LUCID subscales of “thought” and “dissociation,” reflecting higher scores for meditators. The first refers to logical thought about other dream characters (e.g., “while dreaming, I often thought about my own actions”), while “dissociation” refers to experiencing the dream from a third person’s perspective (e.g., “I watched the dream from the outside, as if on a screen”) (Voss et al., 2013). In a similar line, a previous study (García-Campayo et al., 2021) observed that the meditators scored more highly for the “insight” and “dissociation” factors and reported that length of meditation experience was significantly associated with “control” and “dissociation” (i.e., higher scores for more meditation experience). “Dissociation” seems to be clearly related to the mindfulness facet of “observing,” and significant associations were found between these two subscales both in our study and in Garcia-Campayo et al. (2021). While previous studies have indicated that practicing meditation may have a notable influence on lucid dreaming (Baird et al., 2019; Garcia-Campayo, 2022), the lack of significant differences in most of the LUCID subscales in our study leaves this question open.

Finally, the interconnection between the study variables was clear, as were the significant correlations that were observed between most of them and the significant predictive role that many played at least in one of the study outcomes. Two variables were particularly significant in terms of predicting outcomes in our study: on one hand, being a meditator was a significant predictor of higher levels of mindfulness and of having undergone at least one peak experience, which goes in line with the study hypothesis and what previous studies had reported (Falkenström, 2010; Martin et al., 2021). On the other hand, also as expected, absorption was a very relevant variable since it was observed to play a significant predictive role in many outcomes: having a peak experience, non-dual embodiment levels, mindfulness levels, and dream lucidity. Previous studies had already observed significant associations of absorption with transcendent or peak experiences (Glicksohn and Barrett, 2003; Bresnick and Levin, 2006; Studerus et al., 2012), meditation (Holzel and Ott, 2006; Bowden et al., 2012; Grant et al., 2013; Berkovich-Ohana and Glicksohn, 2017), and lucid dreams (Glicksohn and Barrett, 2003; Hunt, 2007; Terhune, 2009; Rosen et al., 2017; Glicksohn, 2019), and therefore our results add to the existing evidence regarding the significant associations between these variables.

### Limitations

The results of this study need to be interpreted in light of several limitations. First, the nature of the relations reported here cannot be attributed to causality. The results are based on self-reported measures, and some bias is expected. The questionnaires used are validated measures that have been consistently utilized in previous studies, and the Spanish adaptation was used in all cases except for the NETI, whose Spanish version has not been validated yet. On the other hand, constructs such as “peak experience” are still hard to measure and, therefore, *ad hoc* questions needed to be included to complement the information assessed by the validated scales, and some of these questions may not have been explicit enough (e.g., “intensity” of the peak experience). Furthermore, it should be pointed out that the LUCID scale does not assess the frequency of lucid dreams but the characteristics of one recent dream, which did not allow this study to draw conclusions on how much more likely meditators were to have lucid dreams compared with non-meditators. Future studies should consider the inclusion of *ad hoc* questions in this regard.

On the other hand, the study used a convenience sample of the general Spanish population that cannot be considered representative of the general population. Further studies should replicate our findings using more heterogeneous samples. Moreover, the classification of the participants as “meditators” not only included frequent meditators but also those who practiced “at least once a month.” It is likely that this subgroup was too heterogeneous and not representative of actual meditators, in which case further studies should consider using a stricter criterion to define meditators. Finally, the exploratory nature of this study implies that our results need to be interpreted with caution; the potential predictive role of the variables included here should be tested in future studies with adequate approaches.

## Conclusion

Our results suggest that having undergone a peak experience is more common among people who practice meditation, even if the frequency of practice is low, compared with those who have never meditated. The number of peak experiences and their intensity, and the ability to self-induce them does not seem to be affected by the practice of meditation; however, some key variables such as non-dual awareness, mindfulness facets, and absorption were significantly higher in meditators. Dream lucidity, on the other hand, was not clearly higher in individuals who practiced meditation as only two subscales (“thought” and “dissociation”) presented significant differences. The interconnection between the study variables was clear, as were the significant correlations that were observed between most of them and the predictive role that many played in at least one study outcome. These exploratory results support the potential of meditation to facilitate transcendent experiences and the wellbeing associated with them, while its effect on dream lucidity remains unclear.

## Data Availability Statement

The data that support the findings of this study are available from the corresponding author upon reasonable request.

## Ethics Statement

The studies involving human participants were reviewed and approved by Comité de Ética de la Investigación de la Comunidad de Aragón (CEICA). The patients/participants provided their written informed consent to participate in this study.

## Author Contributions

JG-C: conceptualization, methodology, resources, writing—review and editing, supervision, and project administration. RH-A: writing—review and editing. AB-S: formal analysis, investigation, data curation, writing—review and editing. SF-M: conceptualization and investigation. RA: conceptualization, writing—review and editing. AP-A: methodology, formal analysis, writing—original draft. All authors contributed to the article and approved the submitted version.

## Conflict of Interest

The authors declare that the research was conducted in the absence of any commercial or financial relationships that could be construed as a potential conflict of interest.

## Publisher’s Note

All claims expressed in this article are solely those of the authors and do not necessarily represent those of their affiliated organizations, or those of the publisher, the editors and the reviewers. Any product that may be evaluated in this article, or claim that may be made by its manufacturer, is not guaranteed or endorsed by the publisher.
