# Supplementary material for: Examining the Relation Between Practicing Meditation and Having Peak Experiences and Lucid Dreams. A Cross-Sectional Study
Source: Front Psychol. 2022 Apr 26;13:858745. doi: 10.3389/fpsyg.2022.858745 (PMC9087568; doi:10.3389/fpsyg.2022.858745)
Supplement: Supplementary file 1 [file Table_1.DOCX]

Supplementary table: bivariate correlations between the study variables.

|  | 1.1 | 1.2 | 1.3 | 1.4 | 1.5 | 2 | 3 | 4 | 4.1 | 4.2 | 4.3 | 4.4 | 4.5 | 4.6 | 4.7 | 4.8 |
| --- | --- | --- | --- | --- | --- | --- | --- | --- | --- | --- | --- | --- | --- | --- | --- | --- |
| 1. FFMQ-total | **.54**  *<.001* | **.66**  *<.001* | **.75**  *<.001* | **.71**  *<.001* | **.72**  *<.001* | **.59**  *<.001* | **.31**  *<.001* | .05  *.488* | .03  *.661* | .04  *.581* | .11  *.079* | **.18**  *.007* | .09  *.160* | .03  *.682* | **-.21**  *.001* | .01  *.945* |
| 1.1. Observing | - | **.21**  *.001* | **.29**  *<.001* | **.16**  *.014* | **.34**  *<.001* | **.30**  *<.001* | **.58**  *<.001* | .13  *.046* | .04  *.523* | .11  *.102* | .14  *.032* | .06  *.342* | .09  *.154* | .23  *<.001* | -.03  *.705* | .03  *.632* |
| 1.2. Describing |  | - | **.35**  *<.001* | **.27**  *<.001* | **.36**  *<.001* | **.35**  ***<.001*** | **.23**  *<.001* | .05  *.465* | -.02  *.771* | -.01  *.893* | .09  *.190* | **.19**  *.003* | .06  *.356* | .00  *.984* | -.03  *.670* | -.03  *.652* |
| 1.3. Acting with awareness |  |  | - | **.46**  *<.001* | **.41**  *<.001* | **.34**  *<.001* | .13  *.047* | -.02  *.807* | -.00  *.980* | -.03  *.679* | .09  *.177* | .05  *.445* | .07  *.299* | -.02  *.761* | **-.18**  *.006* | -.04  *.543* |
| 1.4. Nonjudging |  |  |  | - | **.40**  *<.001* | **.41**  *<.001* | -.02  *.761* | -.10  *.117* | -.01  *.866* | -.04  *.501* | -.05  *.473* | .12  *.058* | -.04  *.507* | -.12  *.066* | **-.26**  *<.001* | -.08  *.221* |
| 1.5. Nonreacting |  |  |  |  | - | **.61**  *<.001* | **.24**  *<.001* | **.15**  *.023* | .11  *.089* | .14  *.032* | **.16**  *.012* | **.16**  *.017* | **.17**  *.009* | .07  *.297* | **-.20**  *.002* | **.18**  *.007* |
| 2. NETI |  |  |  |  |  | - | **.36**  *<.001* | **.19**  *.004* | **.17**  ***.010*** | **.20**  ***.003*** | .10  .129 | **.15**  *.022* | **.18**  *.006* | .09  *.188* | -.10  *.121* | **.21**  *.001* |
| 3. TAS |  |  |  |  |  |  | - | **.32**  *<.001* | **.21**  *.001* | **.27**  ***<.001*** | **.27**  ***<.001*** | .13  *.050* | **.27**  ***<.001*** | **.22**  ***.001*** | .08  *.212* | **.21**  ***.001*** |
| 4. LUCID-total |  |  |  |  |  |  |  | - | **.69**  *<.001* | .**73**  *<.001* | **.75**  *<.001* | **.50**  *<.001* | **.82**  *<.001* | **.65**  *<.001* | **.37**  *<.001* | **.64**  *<.001* |
| 4.1. Insight |  |  |  |  |  |  |  |  | - | **.65**  *<.001* | **.38**  *<.001* | **.14**  *.030* | **.54**  *<.001* | **.47**  *<.001* | .08  *.239* | **.38**  *<.001* |
| 4.2. Control |  |  |  |  |  |  |  |  |  | - | **.40**  *<.001* | .10  *.131* | **.55**  *<.001* | **.53**  *<.001* | .13  *.054* | **.45**  *<.001* |
| 4.3. Thought |  |  |  |  |  |  |  |  |  |  | - | **.35**  *<.001* | **.64**  *<.001* | **.42**  *<.001* | **.26**  *<.001* | **.36**  *<.001* |
| 4.4. Realism |  |  |  |  |  |  |  |  |  |  |  | - | **.38**  *<.001* | **.17**  *.011* | **.09**  *.165* | **.34**  *<.001* |
| 4.5. Memory |  |  |  |  |  |  |  |  |  |  |  |  | - | **.45**  *<.001* | **.29**  *<.001* | **.40**  *<.001* |
| 4.6. Dissociation |  |  |  |  |  |  |  |  |  |  |  |  |  | - | .03  *.604* | **.32**  *<.001* |
| 4.7. Negative emotion |  |  |  |  |  |  |  |  |  |  |  |  |  |  | - | -.04  *.508* |
| 4.8. Positive emotion |  |  |  |  |  |  |  |  |  |  |  |  |  |  |  | - |

***Note***: In *italics*, *p* values. In **bold**, statistically significant values after applying the Benjamini-Hochberg correction.
