# Supplementary material for: Examining the Relation Between Practicing Meditation and Having Peak Experiences and Lucid Dreams. A Cross-Sectional Study
Source: Front Psychol. 2022 Apr 26;13:858745. doi: 10.3389/fpsyg.2022.858745 (PMC9087568; doi:10.3389/fpsyg.2022.858745)
Supplement: Supplementary file 2 [file Table_2.DOCX]

Supplementary material: scales used for obtaining the sociodemographic, meditative and peak experiences information.

**Sociodemographic information:**

Date: _ _ /_ _ /_ _ _ _

Place of residence: ________________________

Sex:

□ Male

□ Female

Age: __________ years

Nationality:_______________

Civil status:

 Married or partnered

 Single

 Separated Divorced

 Widower

Number of children: __________

Coexistence:

 Own home alone

 Own residence with partner

 Own residence with partner and/or children

 Home of relatives

 Address of neighbors or friends

 Residence

 Others (specify):_______________________

Level of studies:

 No education but knows how to read and write

 School graduate (EGB, ESO, FP I, Middle Grade)

 Secondary studies (BUP, higher baccalaureate, COU, PREU, FP II, higher degree)

 University studies

 Others (specify):_______________________

Employment situation:

 Student

 Housewife

 Unemployed With subsidy

 Unemployed without subsidy

 Employee

 Employed but on sick leave (ILT)

 Retired

 Disabled permanent disability

 Other (specify): _________________

Type of employment contract (employees only)

 Permanent civil servant

 Interim civil servant

 Indefinite contract

 Temporary contract of less than 6 months

 Temporary contract of more than 6 months

 Temporary contract without specifying the duration (work and service, or similar)

 Worker of a temporary employment agency (ETT)

 Work without contract

 Work on your own (self-employed, entrepreneurs, liberal professionals)

 Other contractual relationship (specify):

**Information on type of meditative practice:**

We would like to know exactly what meditation techniques you practice, and how often, to better understand what specific effects each type of meditation produces. Below, we describe the most common meditation techniques:

| 1. **Concentrative or focused attention (FA):** It is based on staying focused on breathing or another single object of attention. Examples: mindfulness of breath, breath counting, body awareness practices, mantras, samatha or supported calming of mind (e.g. breath anchoring), etc. |
| --- |
| 1. **Contemplation or open monitoring (OM):**  It consists of paying attention and being aware of everything that appears and passes through consciousness, without selecting a particular focus of attention. OM can be object-oriented (directing attention to thoughts, perceptions, and sensations that appear in consciousness) or person-oriented (sustained recognition of the quality of attention as such developed by the individual). Examples: objectless choice meditation, mindfulness cultivation (Greco-Roman philosophy), Samatha practices, or unsupported calm abiding. |
| 1. **Compassion or loving kindness:** It consists of generating, either through images or phrases, feelings of compassion, self-compassion or loving kindness. |
| 1. **Values:** Seeks to consolidate an ethical framework and restructure values and priorities, achieving a reorientation towards what is truly important in life for each one. |
| 1. **Deconstructive:** They use self-questioning to develop self-knowledge regarding the nature and dynamics of conscious experience. They may be object-oriented, investigating the objects of consciousness; subject-oriented, exploring the nature of thoughts, perception, and other affective and cognitive processes; or non-dual, focused on undoing the reification or reification of a separate witness “observer” of the objects of consciousness. Examples: Self-inquiry, noticing sensations or breathing and seeing how they continually change, or dissecting thoughts and emotions into their different component parts. |
| 1. **Informal mindfulness practices:** Do daily activities mindfully. |

**For each of the meditations, please indicate the percentage of time you currently spend on each of these techniques based on the total time you spend on meditation.** Note: If any of these do not fit the practices, assign a value of 0.

For example: Focused Attention 30% of my time, Open Monitoring 40%, Compassion 20%, Values 5%, Deconstructive 5% (must add up to 100%).

|  | % dedicated to each meditation |
| --- | --- |
| Concentrative or focused attention (FA) |  |
| Contemplation or open monitoring (OM) |  |
| Compassion or loving kindness |  |
| Values |  |
| Deconstructivive |  |
| Informal mindfulness practices |  |
| Total | 100 % |

**Please indicate how often you currently practice each technique (approximate).** Note: Mark “never” if you do not practice any of the meditative practices.

|  | Daily | Between 3 and 4 times a week | Once a week | Between 2 and 3 times a month | Occasionally | Never |
| --- | --- | --- | --- | --- | --- | --- |
| Concentrative or focused attention (FA) |  |  |  |  |  |  |
| Contemplation or open monitoring (OM) |  |  |  |  |  |  |
| Compassion or loving kindness |  |  |  |  |  |  |
| Values |  |  |  |  |  |  |
| Deconstructivive |  |  |  |  |  |  |
| Informal mindfulness practices |  |  |  |  |  |  |

**Currently, when you meditate, how long are you practicing on average in each of these practices? (answers in minutes).** Note: Assign a value of 0 if you do not practice any of the meditative practices.

|  | Minutes |
| --- | --- |
| Concentrative or focused attention (FA) |  |
| Contemplation or open monitoring (OM) |  |
| Compassion or loving kindness |  |
| Values |  |
| Deconstructivive |  |
| Informal mindfulness practices |  |

**How long have you been practicing each of these techniques?** Note: Check the “never” option if you do not know or have never practiced this type of meditation.

|  | Never | Less than 1 year | Between 1 and 3 years | 4-6 years | 7-9 years | More than 10 years |
| --- | --- | --- | --- | --- | --- | --- |
| Concentrative or focused attention (FA) |  |  |  |  |  |  |
| Contemplation or open monitoring (OM) |  |  |  |  |  |  |
| Compassion or loving kindness |  |  |  |  |  |  |
| Values |  |  |  |  |  |  |
| Deconstructivive |  |  |  |  |  |  |
| Informal mindfulness practices |  |  |  |  |  |  |

**Then, if you want, you can expand or comment on your meditative technique.**

**Evaluation of Peak Experiences:**

A Peak or Summit experience is defined as:

“A state of unity with mystical characteristics, an experience in which time tends to vanish and the overwhelming feeling makes it seem that all needs are fulfilled”

In other words, they are the especially happy, fulfilling and exciting moments in the life of any individual. And, among the main characteristics that describe the nature of this type of experience, the following stand out, among others:

**• What is perceived is captured completely and exclusively, with total attention. The very perception seems to be enriched.**

**• There is a characteristic disorientation with respect to time and space.**

**• Transcendence of the ego. The person ignores her own interests and may be altruistic. Perception is more centered on the object than on the ego.**

**• It is an experience that cannot be mastered. It is the experience that comes to the person. Therefore, the will remains in expectation, receiving without demanding.**

**• There is an emotional reaction of admiration, awe, reverence and humility before the experience.**

**• There is a complete loss, although temporary, of all fear, defense, anxiety, inhibition and control, ceasing the renunciations, delays and coercions.**

**• They are transitory moments, instants with an intense sensation of positive affection, freedom and fullness.**

1. Have you had one or more experiences that meet these characteristics throughout your life?

*Yes / No*

1. If you have marked “Yes” in the previous question, How many times in your life have you had this kind of experience?
2. At what ages?
3. What do you consider to have been the average intensity of these experiences? Indicates a numerical value between 0 (no intensity and 10 (maximum intensity)
4. Under what circumstances have they taken place? (describe in several lines)
5. Next, summarize in a maximum of 20 lines, the peak experience that you consider most important.
6. Have those experiences changed your life? (score from 0= not at all to 10= maximum)
7. How have those peak experiences changed you?
8. What about the following options? (Indicate all that you consider)

- Life's sense

- Relationship with other people

- Regulation of emotions

- Spiritual Quest

1. Have you ever self-induced (performed an activity to achieve it) those experiences?

*Yes / No*

How?

1. Do you think you could self-induce these experiences?

*Yes (How?)*

*No*

*I don’t know (explain it)*

1. Of the following options, which ones appeared or usually appear?

*List with the main characteristics of the PICO experiences:*

- Total attention focused on the present.

- Intense positive affect (feeling of well-being and fullness).

- Admiration, astonishment, reverence and humility before the experience.

- Transcendence of the ego.

- Complete loss, although temporary, of all fear, defense, anxiety, inhibition and control, ceasing resignations, delays and coercion.

- Disorientation with respect to time and space.

1. Of the above, if you had to choose one, which one predominated?
